# MicroRNAs control the apoptotic threshold in primed pluripotent stem cells through regulation of BIM

**DOI:** 10.1101/gad.245621.114

**Published:** 2014-09-01

**Authors:** Barbara Pernaute, Thomas Spruce, Kimberley M. Smith, Juan Miguel Sánchez-Nieto, Miguel Manzanares, Bradley Cobb, Tristan A. Rodríguez

**Affiliations:** 1 British Heart Foundation Centre for Research Excellence, National Heart and Lung Institute, Imperial Centre for Translational and Experimental Medicine, Imperial College London, London W12 0NN, United Kingdom;; 2 Royal Veterinary College, London NW1 0TU, United Kingdom;; 3 Department of Cardiovascular Developmental and Repair, Centro Nacional de Investigaciones Cardiovasculares-CNIC, 28029 Madrid, Spain

**Keywords:** epiblast, pluripotency, apoptosis, microRNAs, Bim, Dicer

## Abstract

Mammalian primed pluripotent stem cells have been shown to be highly susceptible to cell death stimuli due to their low apoptotic threshold, but how this threshold is regulated remains largely unknown. Here we identify microRNA (miRNA)-mediated regulation as a key mechanism controlling apoptosis in the post-implantation epiblast. Moreover, we found that three miRNA families, miR-20, miR-92, and miR-302, control the mitochondrial apoptotic machinery by fine-tuning the levels of expression of the proapoptotic protein BIM. These families therefore represent an essential buffer needed to maintain cell survival in stem cells that are primed for not only differentiation but also cell death.

## Abstract (precis)

Pernaute et al. identify miRNA-mediated regulation as a key mechanism controlling apoptosis in the post-implantation epiblast. Three miRNA families, miR-20, miR-92, and miR-302, control the mitochondrial apoptotic machinery by fine-tuning the levels of expression of the proapoptotic protein BIM. These miRNA families are needed to maintain cell survival in stem cells that are primed for not only differentiation but also cell death.

In the early mammalian embryo, two different states of pluripotency can be distinguished: a naïve state present in the epiblast of the preimplantation embryo and in mouse embryonic stem cells (mESCs) and a state primed for differentiation present in the epiblast of the post-implantation embryo and in epiblast stem cells (EpiSCs) (Nichols and Smith 2009). These two stages differ in not only their gene expression profiles, epigenetic marks, and metabolism but also the signaling pathways that maintain them. Naïve pluripotent cells rely on inhibition of ERK1/2 and GSK3 and activation of JAK/STAT signaling, whereas primed pluripotent cells depend on Activin and FGF signaling. Remarkably, the majority of human ESC (hESC) lines derived to date display primarily primed pluripotent state features, suggesting that they are more similar to EpiSCs than to mESCs (Pera and Tam 2010). One of these features is a hypersensitivity to DNA damage due to a high priming of their apoptotic machinery (Heyer et al. 2000; Liu et al. 2013). Although the regulation of naïve pluripotency has been extensively studied in mESCs, the regulatory networks governing primed pluripotent stem cells are only starting to be elucidated, and little is known regarding the mechanisms controlling their susceptibility to cell death stimuli.

MicroRNAs (miRNAs) are ∼22-nucleotide (nt)-long RNAs that specifically bind to target mRNAs and impair their translation, often leading to the mRNAs’ subsequent degradation. miRNAs have an essential role in multiple processes, including development, maintenance of tissue homeostasis, and disease. Most miRNA target recognition occurs through perfect matching between the second-eighth nucleotide of the miRNA, named the miRNA seed sequence, and the 3′ untranslated region (UTR) of the target mRNA (Bartel 2009). Accordingly, miRNAs with the same seed sequence share most of the same targets and can be functionally grouped into miRNA seed families.

The importance of miRNAs during early mouse development is highlighted by the strong phenotypes associated with the deletion of genes essential for miRNA synthesis, such as *Dicer*, *Dgcr8*, and *Ago2*; in all cases, gene loss results in embryonic lethality by ∼7.5 d post-coitum (dpc) (Bernstein et al. 2003; Morita et al. 2007; Wang et al. 2007). Interestingly, miRNAs are dispensable for mouse preimplantation development (Suh et al. 2010), but at the post implantation stage, their absence (due to knockout of *Dicer*) results in a massive increase in cell death in the epiblast (Spruce et al. 2010). Together, these observations suggest a specific requirement for miRNAs in the maintenance of primed pluripotent stem cell survival.

Here, by miRNA expression profiling of mouse embryos from 5.5 to 8.5 dpc, we identify four miRNA seed families that account for the majority of miRNA expression during early development. Analysis of the effects of *Dicer* deletion in EpiSCs and embryos indicates that three of these families exert the highest impact on the transcriptome of primed pluripotent stem cells and control the apoptotic threshold in these cells by regulating the levels of expression of the proapoptotic protein BIM. Furthermore, we found that the extensive cell death in the epiblast induced by loss of miRNAs is precluded by concurrent loss of *Bim*, confirming that this protein is the main target of miRNAs regulating cell survival during early embryogenesis.

## Results and Discussion

### Dynamic changes in miRNA expression accompany changes in the pluripotent state

A number of studies have characterized the miRNA expression profile of hESCs and mESCs as well as preimplantation mouse embryos (Tang et al. 2007, 2010; Babiarz et al. 2008; Yang et al. 2008; Leung et al. 2011). However, to date, no global analysis of miRNA expression has been performed on primed pluripotent cells in vivo. As a first step toward understanding miRNA function in the post-implantation epiblast, we carried out a quantitative PCR (qPCR)-based profiling of 312 well-characterized miRNAs in pools of whole embryos at 5.5 dpc, 6.5 dpc, 7.5 dpc, and 8.5 dpc (Supplemental Table S1) and compared these expression profiles with a previously published qPCR-based miRNA profiling done on the 3.5-dpc inner cell mass (ICM) (Tang et al. 2010). The expression pattern of the most abundant miRNAs was then established by in situ hybridization.

**Figure 1.**
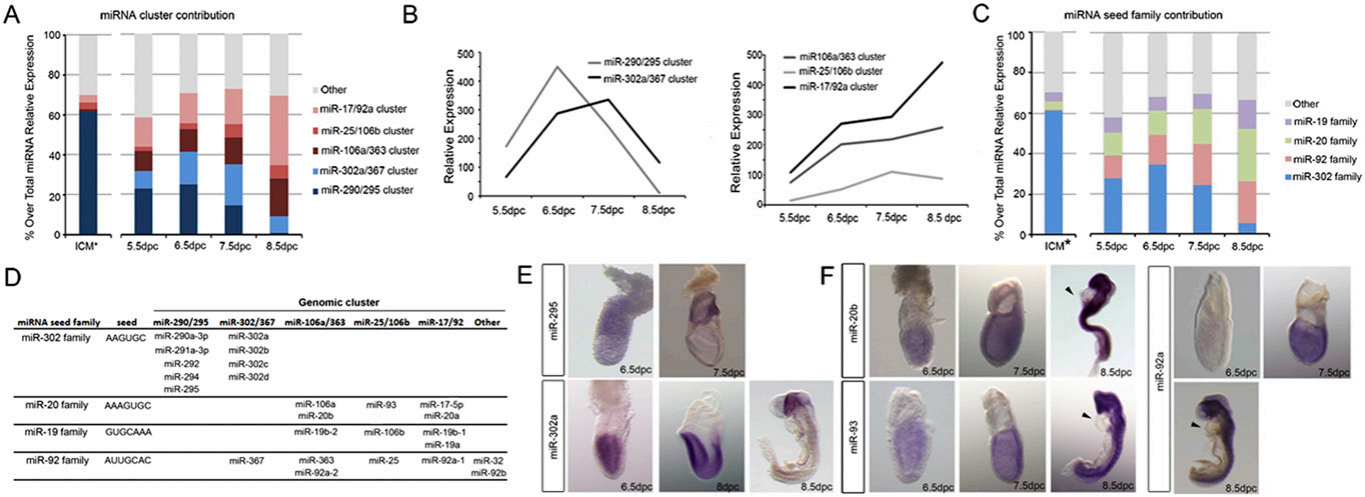
Four miRNA families account for most miRNA expression during early post-implantation development. (*A*,*B*) Relative contribution (*A*) and relative expression (*B*) of highly expressed miRNA clusters at each developmental stage. (*C*,*D*) Relative contribution (*C*) and genome distribution (*D*) of highly expressed miRNA seed families at each developmental stage. (*E*,*F*) In situ hybridization of miRNAs located in each highly expressed cluster. The star indicates data obtained from Tang et al. (2010). Arrows point to lack of miRNA expression in the heart.

These studies found that the 30 most abundant miRNAs at each developmental stage accounted for 77%–85% of the total detected miRNA expression (Supplemental Fig. S1A). Most of these miRNAs are expressed from just five miRNA clusters (Fig. 1A,D). The miR-290/295 cluster accounts for the majority of miRNA expression in ESCs and in the preimplantation epiblast (ICM) (Fig. 1A; Tang et al. 2010). However, in accordance with recent findings, we saw that, upon implantation, a switch occurs, and expression of miRNAs of the miR-290/295 cluster becomes restricted to the extraembryonic trophoblast (Fig. 1E; Parchem et al. 2014), and expression of miRNAs from the miR-302/367, miR-25/106b, miR-17/92a, and miR-106a/363 clusters becomes restricted to the epiblast (Fig. 1E,F). As development proceeds, expression from the miR-25/106b, miR-17/92a, and miR-106a/363 clusters increases and remains restricted to the embryonic region, whereas expression of those miRNAs expressed from the miR-302/367 becomes localized to the anterior neural tube by 8.5 dpc (Fig. 1B,E,F).

Four miRNA seed families are encoded by the clusters highly expressed in the post-implantation epiblast and therefore account for the majority of miRNA expression observed there: the miR-19, miR-20, miR-92, and miR-302 families (Fig. 1C,D). The miR-302 family plays a role in the control of pluripotency, differentiation, and cell cycle progression in mESCs and hESCs (Card et al. 2008; Sinkkonen et al. 2008; Wang et al. 2008; Rosa et al. 2009). In line with its role in stem cell pluripotency, members of the miR-302 seed family are found within the miR-290/295 and miR-302/367 clusters and are specifically expressed at the naïve and primed pluripotent stages, respectively, both in vitro (Jouneau et al. 2012) and in the embryo (Fig. 1E). The miR-19, miR-20, and miR-92 families are encoded by the miR-106a/363, miR-17/92, and miR-25/106b clusters (with the exception of miR-367, which is located within the miR-302/367 cluster) (Fig. 1D). These clusters showed widespread expression in embryonic tissues at early post-implantation stages, except for the heart at 8.5 dpc (Fig. 1F; Supplemental Fig. S1B), suggesting a role in the control of tissue homeostasis.

Together, these data show that four miRNA families found within five miRNA clusters account for most miRNA expression in the primed pluripotent stage in the mouse embryo in vivo. Interestingly, orthologous miRNA families are expressed early in development in other vertebrates, suggesting that they play a conserved role at these stages (Giraldez et al. 2005; Rosa et al. 2009).

### The miR-20, miR-92, and miR-302 miRNA seed families regulate survival of primed pluripotent stem cells

**Figure 2.**
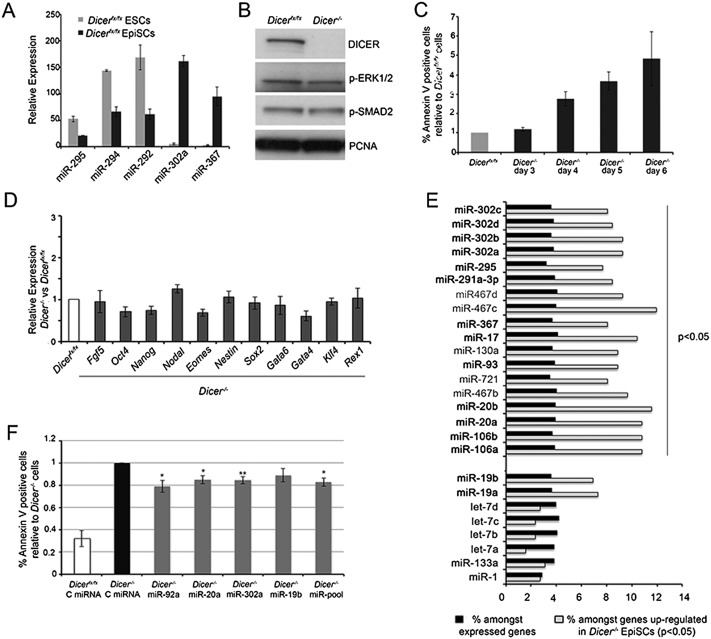
The miR-20, miR-92, and miR-302 seed families exert a high impact on EpiSC transcriptome and cell survival. (*A*) Switch in miRNA expression from the miR-290/295 to miR-302/367 cluster between ESCs and EpiSCs. Average from three ESCs and EpiSCs samples ±SEM is shown. (*B*) Loss of DICER protein and correct activation of FGF and Activin signaling pathways in EpiSCs at day 5 after *Dicer* deletion. (*C*) Increase in cell death is observed in EpiSCs from day 4 after *Dicer* deletion. Average of four experiments ±SEM is shown. (*D*) qPCR data showing expression of pluripotency and lineage-specific markers in *Dicer*-deleted compared with *Dicer*^*fx/fx*^ EpiSCs. Average fold change from four experiments ±SEM is shown. (*E*) miRNAs showing a significant enrichment in predicted targets among genes up-regulated in *Dicer*^*−/−*^ EpiSCs. miRNAs belonging to the miRNA seed families highly expressed in the embryo are in bold. miRNAs not expressed in the early embryo (let-7, miR-1, and miR-133) are shown as negative control. (*F*) Transfection of miRNA mimics representative of each highly expressed miRNA family or a combination of representative members of each family reduces the levels of cell death in *Dicer*-deleted EpiSCs. Cells were transfected with miRNA mimics or a control miRNA at days 2 and 4 and were FACS-analyzed at day 6 after *Dicer* deletion. Average of six experiments ±SEM are shown. Student’s *t*-test: (*) *P* < 0.05; (**) *P* < 0.01.

Many studies have addressed miRNA function in the naïve state of pluripotency (mostly in mESCs); however, little is known regarding the role of these regulators in the primed pluripotent state. We previously described an essential role for *Dicer* in the control of cell survival in the epiblast at post-implantation stages (Spruce et al. 2010). To study the mechanism underlying this regulation, we established a tamoxifen-inducible *Dicer* conditional EpiSC line (*Dicer*^*fx/fx*^) by direct differentiation from *Dicer*^*fx/fx*^ ESCs (Fig. 2A; Supplemental Fig. S2A,B; Nesterova et al. 2008; Guo et al. 2009). This model permitted efficient *Dicer* deletion and miRNA depletion within a short window of time (Fig. 2B; Supplemental Fig. S2C,D), allowing us to pinpoint the primary requirements for miRNAs in EpiSCs. Mimicking what occurs in the embryo (Spruce et al. 2010), the main phenotype observed in these cells after miRNA loss was a dramatic increase in apoptosis, as measured by Annexin V and cleaved Caspase 3 staining (Fig. 2C; Supplemental Fig. S2E). This cell death was not accompanied by any change in the expression of pluripotency or lineage-specific markers (Fig. 2D) or in the activation of the signaling pathways (FGF and Activin) required for EpiSC maintenance (Fig. 2B), indicating that regulation of cell survival constitutes the primary role of miRNAs in the primed pluripotent state.

To identify key mRNA–miRNA interactions that mediate the cell death phenotype, we first compared the gene expression profile of *Dicer*-deleted with undeleted EpiSCs by microarray analysis. A total of 509 genes were misregulated upon miRNA loss, 261 of which were up-regulated by at least 1.2-fold in *Dicer*-deleted cells. The degree to which these up-regulated genes are regulated by miRNAs was analyzed using the data analysis platform Babelomics (Medina et al. 2010), which tests for enrichment for miRNA targets. The results from this analysis showed a significant enrichment for targets of 18 miRNAs (Fig. 2E). Thirteen of these belong to three of the four miRNA seed families previously found to be highly expressed in the early post-implantation embryo: miR-20, miR-92, and miR-302 (Fig. 1C). Although the enrichment for targets of the miR-19 family is higher than that of miRNAs expressed in differentiated tissues (such as let-7 or miR-1), it is not statistically significant. Of the remaining five miRNAs, miR-130a and miR-721 share the same seed sequence, and miR-130a is among the 30 most highly expressed miRNAs in the early embryo (Supplemental Fig. S1A). Finally, miR-467a, miR-467b, and miR-467c share the same hexamer seed sequence (AAGUGC) as the miR-302 family and have been predicted to regulate the same processes (Zheng et al. 2011b); however, their expression was restricted to the extraembryonic trophoblast at post-implantation stages (Supplemental Fig. S2F). No significant enrichment for miRNA targets was found when analyzing the genes down-regulated in *Dicer*^*−/−*^ EpiSCs, suggesting that the Babelomics miRNA target predictions represent true interactions. Overall, these data show that the miRNA families that are most abundant in the post-implantation embryo exert the highest impact on the EpiSC transcriptome and suggest that these families are responsible for the defects observed in the epiblast of *Dicer*^−/−^ embryos.

We next tested whether restoring the expression of these miRNA families would reduce the cell death observed in *Dicer*-deleted cells. Transfection of *Dicer*^*−/−*^ EpiSCs with the highest expressed members of each of these families or a combination of them led to a reduction in the amount of cell death observed in mutant cells (Fig. 2F). miR-92a caused the strongest reduction in apoptosis, whereas the decrease observed after miR-19b transfection was not statistically significant, suggesting a smaller contribution of this miRNA family to the control of EpiSC survival. A prosurvival function for miRNAs belonging to these four families has been described both in ESCs exposed to genotoxic stress and during late development and oncogenesis (Mendell 2008; Ventura et al. 2008; Zheng et al. 2011a), suggesting that they may have a general anti-apoptotic function.

### miRNAs regulate cell death through the mitochondrial apoptotic machinery in EpiSCs

**Figure 3.**
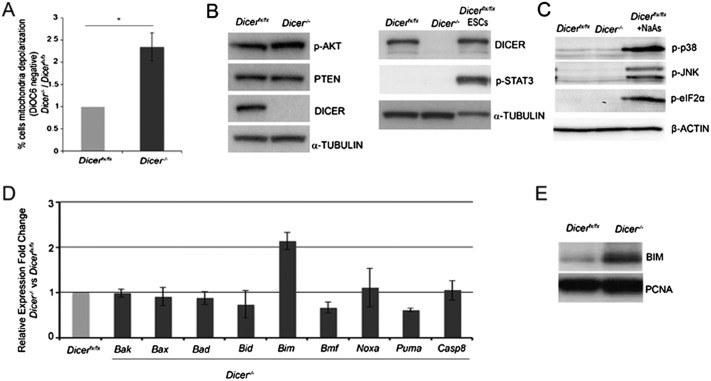
miRNAs control apoptosis through the mitochondrial apoptotic machinery and lead to BIM up-regulation in EpiSCs. (*A*) Increased DiOC6 staining in *Dicer*-deleted EpiSCs 5 d after tamoxifen treatment. Average of three experiments ±SEM is shown. (*B*,*C*) *Dicer*-deleted EpiSCs show normal levels of activation of the prosurvival signaling pathways JAK/STAT and AKT and of the AKT inhibitor PTEN (*B*) and no activation of proapoptotic and stress response pathways (*C*). (*D*) qPCR showing expression of proapoptotic factors involved in the mitochondrial apoptotic pathway in *Dicer*^*fx/fx*^ and *Dicer*^*−/−*^ EpiSCs. (*E*) Western blot showing an increase in BIM expression in *Dicer*-deleted EpiSCs. All analyses were performed at day 5 after *Dicer* deletion. *Dicer*^*fx/fx*^ ESCs and *Dicer*^*fx/fx*^ EpiSCs treated with 60 μM stressing agent sodium arsenite were used as a positive control. Average fold change from three experiments ±SEM is shown. Student’s *t*-test: (*) *P* < 0.05.

In order to identify the key miRNA targets regulating cell survival during early development, we set out to establish the type of cell death induced by miRNA depletion. Addition of the pan-caspase inhibitor Z-VAD-FMK to *Dicer*^*−/−*^ EpiSCs completely rescued their survival defect (Supplemental Fig. S3A), pointing to a caspase-dependent type of cell death. Caspase-dependent cell death is known as apoptosis and can be induced by two different pathways. The mitochondrial pathway results in mitochondrial membrane depolarization and cytochrome c release to the cytosol, which triggers the activation of effector caspases (Caspase 3 and Caspase 7). In contrast, the nonmitochondrial pathway relies primarily on direct activation of effector caspases by Caspase 8 independently of the mitochondria (Tait and Green 2010). We observed both mitochondrial membrane depolarization and cytochrome c release after *Dicer* deletion in EpiSCs (Fig. 3A; Supplemental Fig. S3B), indicating that miRNAs are required in the primed pluripotent state to control apoptosis occurring via the mitochondrial pathway.

Activation of the mitochondrial apoptotic machinery mostly depends on the balance between pro- and anti-apoptotic members of the Bcl2 protein family. Various signaling pathways have a prosurvival or proapoptotic effect depending on the regulation that they exert over the balance of Bcl2 family members. In miRNA-depleted EpiSCs, we observed no difference in the activation of proapoptotic (p38, JNK, ERK, and SMAD2) or anti-apoptotic (STAT3 and AKT) signaling pathways or in the expression of the AKT inhibitor PTEN (Fig. 3B,C). Similarly, no activation of the stress response factor eIF2a was seen in mutant cells (Fig. 3C). Consequently, we searched for proapoptotic factors that could be direct miRNA targets among the genes up-regulated in *Dicer*-deleted EpiSCs and found that *Bim* was the only one with significantly increased expression after miRNA depletion (Supplemental Fig. S3C). This was confirmed by qPCR and Western blot analysis of proapoptotic factors in *Dicer*^*fx/fx*^ and *Dicer*-deleted cells (Fig. 3D,E; Supplemental Fig. S3D). The expression levels of anti-apoptotic family members were found to be unchanged after *Dicer* deletion, ruling out an indirect effect of miRNA loss leading to their down-regulation (Supplemental Fig. S3E). This points to increased BIM expression as the primary cause for the apoptosis induced by miRNA depletion in the primed pluripotent state.

### BIM is the key miRNA target regulating the apoptotic threshold in primed pluripotent stem cells

**Figure 4.**
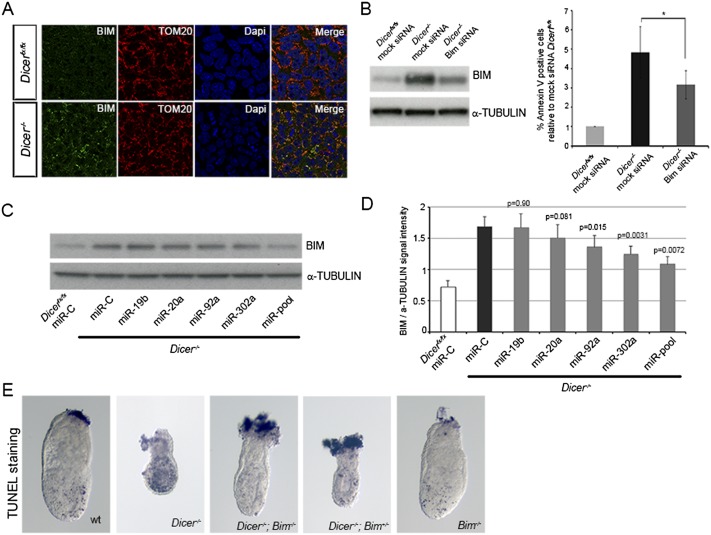
miRNA families highly expressed in the embryo control cell survival through the repression of BIM. (*A*) BIM localization at the mitochondria of *Dicer*^*fx/fx*^ and *Dicer*-deleted EpiSCs. (*B*) Transfection of *Dicer*-deleted EpiSCs with a Bim siRNA reduces the levels of apoptosis in these cells. Cells were transfected with a Bim siRNA or a control siRNA at day 2 and were FACS-analyzed at day 4 after *Dicer* deletion. Average from six experiments ±SEM is shown. Student’s *t*-test: (*) *P* < 0.05. (*C*,*D*) Transfection of miR-20a, miR-92a, and miR-302a reduces the levels of BIM in *Dicer*^*−/−*^ EpiSCs, whereas miR-19b does not affect BIM expression. Cells were transfected with miRNA mimics or a control miRNA at day 2 and analyzed at day 4 after *Dicer* deletion. Average of six experiments ±SEM is shown. *P*-value corresponds to Student’s *t*-test. (*E*) All *Dicer*^*−/−*^*;Bim*^*−/−*^ and three out of six *Dicer*^*−/−*^*;Bim*^*+/−*^ 6.5-dpc embryos show levels of TUNEL staining similar to their wild-type littermates. Embryos representative for the wild-type (*n* = 5), *Dicer*^*−/−*^ (*n* = 3), *Dicer*^*−/−*^*;Bim*^*−/−*^ (*n* = 5), *Dicer*^*−/−*^*;Bim*^*+/−*^ (*n* = 6), and *Bim*^*−/−*^ (*n* = 7) genotypes are shown.

The proapoptotic protein BIM is a known target of the miR-92, miR-19, and miR-20 families in a number of cell types (Koralov et al. 2008; Petrocca et al. 2008; Xiao et al. 2008; Su et al. 2009) and is a predicted target of the miR-302 family (http://www.targetscan.org). We observed that the increase in BIM expression occurred as early as day 4 after the induction of *Dicer* deletion (Supplemental Fig. S4A), which is when the levels of miRNAs decrease and the cells start dying by apoptosis (Fig. 2C; Supplemental Fig. S2D). Importantly, BIM was found in the mitochondria of both wild-type and mutant EpiSCs (Fig. 4A), implying that it does not need to translocate to this organelle in this cell type but is already localized there and possibly kept inactive by its interaction with anti-apoptotic Bcl2 family members.

We next analyzed the effect that BIM knockdown had on *Dicer*-deleted EpiSCs. Cells were transfected with a Bim siRNA at day 2 after the induction of *Dicer* deletion, and the level of BIM expression and its effect on cell survival were analyzed at day 4. We observed a rescue of cell death in these miRNA-depleted cells that was directly proportional to the reduction of BIM levels (Fig. 4B; Supplemental Fig. S4B). We then addressed whether the prosurvival effects that the miR-20a, miR-92a, and miR-302a miRNA families exerted on EpiSCs were mediated by BIM down-regulation. Transfection of representative members of each of these families as well as of a combination of representative members of the miR-19, miR-20, miR-92, and miR-302 families led to a decrease in BIM expression in *Dicer*-deleted EpiSCs (Fig. 4C,D). No reduction in BIM expression was observed after miR-19b transfection, which correlates with the small effect that this miRNA family has on EpiSC survival (Fig. 2F). Altogether, these results indicate that miRNAs have an essential role in maintaining cell survival in EpiSCs and that they primarily do this by repressing the expression of the proapoptotic protein BIM.

Finally, we asked whether BIM misregulation is also the cause of the increase in cell death seen in *Dicer*-deleted embryos. TUNEL staining demonstrated that *Bim* knockout leads to a complete rescue of the cell death phenotype observed in the epiblast of *Dicer*^*−/−*^ embryos in 100% of double mutants (*n* = 5). Furthermore, deletion of only one *Bim* allele rescued the cell survival in three out of six *Dicer*^*−/−*^ embryos (Fig. 4E). The levels of apoptosis seen in the double-knockout embryos and in the rescued *Dicer*^*−/−*^*;Bim*^*+/−*^ embryos were similar to those of wild-type embryos (Fig. 4E), which suggests that the rescue achieved is not due to an absolute requirement for BIM in apoptosis in the embryo but rather the elimination of the apoptosis caused by BIM overexpression after miRNA depletion. It is worth noting that, although *Bim* deletion led to the complete elimination of the excessive cell death seen in *Dicer*^*−/−*^ embryos, they still had an obvious developmental delay and displayed morphological defects. These are most likely due to the requirements of miRNAs for the development of the extraembryonic tissues (Spruce et al. 2010).

miRNAs often exert their regulatory effect by inhibiting multiple targets involved in a process rather than regulating individual proteins. However, here we found that three highly expressed miRNA families control cell survival during early embryogenesis by regulating the expression of a single target: the proapoptotic protein BIM. This role of miRNAs in regulating primed pluripotent stem cell survival may add to the understanding of why miRNA-depleted mESCs fail to differentiate (Kanellopoulou et al. 2005; Murchison et al. 2005; Wang et al. 2007). Given that the first step of differentiation is the transition from the naïve to the primed state of pluripotency, it is likely that miRNA-depleted ESCs undergo apoptosis as they initiate this process, which would contribute to their block in differentiation.

Although little is known regarding the regulation of cell survival in primed pluripotent stem cells, a number of studies suggest these cells present a hypersensitivity to cell death-inducing agents when compared with more differentiated tissues or cell types (Heyer et al. 2000; Liu et al. 2013). This high sensitivity to apoptosis is likely to be associated with a tight regulation of cell death given that these cells have the potential to contribute to all tissues, including the germline. It is tempting to speculate that, by fine-tuning the levels of expression of the proapoptotic protein BIM, miRNAs allow pluripotent stem cells to maintain expression of proapoptotic proteins within a threshold that can be altered rapidly by post-transcriptional mechanisms and in this way allow these cells to respond rapidly and efficiently to cell death signals, making them, in a sense, primed for cell death. Deciphering the regulatory roles of the miRNA families that we identified here will not only give insight into how the apoptotic threshold is established in different developmental contexts but also contribute to our understanding of the role they play in disease.

## Materials and methods

### Mice, whole-mount in situ hybridization (WISH), TUNEL staining, and immunohistochemistry

*Dicer*^*+/−*^ (Cobb et al. 2005), *Bim*^*+/−*^ (Bouillet et al. 1999), and *Dicer*^*+/−*^*;Bim*^*+/−*^ mice were maintained in a mixed background. Wild-type CD1 mice were used to obtain embryos for the miRNA WISH and miRNA qPCR arrays. In all cases, embryos were genotyped after performing the experiments following previously published methods. miRNA WISH was performed in whole embryos using LNA probes (Exiqon) DIG-labeled on the 3′ end following previously described methods, and TUNEL staining in embryos was performed as described elsewhere (Spruce et al. 2010).

### miRNA qPCR and qPCR-based microarray

ESCs, EpiSCs, and embryo samples were used to extract total RNA with the *mir*Vana miRNA isolation kit (Ambion) following the manufacturer's instructions. Pools of ∼200 embryos at 5.5 dpc, 100 embryos at 6.5 dpc, 20 embryos at 7.5 dpc, and 10 embryos at 8.5 dpc from different litters were used for total RNA extraction. From 5.5 to 7.5 dpc, the whole embryo was analyzed, including the extraembryonic regions. TaqMan miRNA probes (Applied Biosystems) were used to perform miRNA qPCRs in stem cells. cDNA was synthetized with the TaqMan miRNA reverse transcription kit (Applied Biosystems), and qPCR for individual miRNAs was performed using TaqMan universal PCR master mix (Applied Biosystems). sno135 and sno202 were used to normalize miRNA expression in ESCs and EpiSCs, respectively. For the qPCR-based microarrays in embryos, total RNA samples were processed and analyzed by Geneservice using the TaqMan mouse miRNA assay set version 1.0 (Applied Biosystems). miRNA expression was normalized against sno202. It remains unclear whether miR-709 and miR-720 are indeed miRNAs (Chiang et al. 2010); therefore, their relative expression was excluded from the data analysis.

### Cell culture and manipulation

*Dicer*^*fx/fx*^ ESCs were maintained and manipulated as detailed in the Supplemental Material. Annexin V and DiOC6 staining, FACS analysis, and EpiSC immunocytochemistry are also detailed in the Supplemental Material.

### Western blot

Laemmli buffer (0.05M Tris-HCl at pH 6.8, 1% SDS, 10% glycerol, 0.001% bromophenol blue, 0.1% b-mercaptoethanol) was used for protein extraction. Western blot was performed following standard procedures. Membrane and cytosolic extracts were separated following previously published methods (Ramsby and Makowski 2011) with minor modifications. Details for the subcellular fractionation procedure and the antibodies used can be found in the Supplemental Material.

### mRNA qPCR and microarrays

Total RNA was isolated using RNeasy (Qiagen). SuperScript III reverse transcriptase (Invitrogen) was used to synthetize cDNA, and SYBR PCR master mix (Qiagen) was used to perform the qPCR reaction. mRNA expression levels were calculated using the comparative Ct value method, and b-Actin was used for normalization. Primer sequences and microarray analysis are detailed in the Supplemental Material.
